# Prospective, Long-Term Functional Outcomes of Extra-Osseous Talotarsal Stabilization (EOTTS) Using HyProCure in Adult Patients with Talotarsal Joint Instability: Assessment of Physical Activity and Patient Satisfaction

**DOI:** 10.3390/jcm12144872

**Published:** 2023-07-24

**Authors:** Łukasz Kołodziej, Dawid Ciechanowicz, Maria Wójtowicz, Marta Król, Małgorzata Szabałowska, Sebastian Kwiatkowski, Mateusz Szymczak, Radomir Czajka

**Affiliations:** 1 Department of Orthopaedics, Traumatology and Musculoskeletal Oncology, Pomeranian Medical University, 71-281 Szczecin, Poland; 2 ST Medical Clinic, 71-004 Szczecin, Poland; 3 Independent Public Healthcare Center, 87-500 Rypin, Poland

**Keywords:** extra-osseous talotarsal stabilization, talotarsal joint instability, HyProCure, physical activity, satisfaction

## Abstract

Background: The partial dislocation of the talus from the calcaneus and navicular bones is a primary factor leading to a prolonged overpronation during weightbearing. This study aimed to assess the possibility of returning to physical activity and long-term patient satisfaction after an extra-osseous talotarsal stabilization (EOTTS) procedure with a HyProCure sinus tarsi implant for partial talotarsal joint dislocation (TTJ). Methods: A total of 41 adult patients (61 feet), with an average age of 46.41, were included and treated surgically with EOTTS as a stand-alone surgery. Physical activity and functional scores were assessed pre- and post-operatively using questionnaires—the UCLA Activity Score, Symptom-Related Ankle Activity Scale (SAAS), Sports Frequency Score (SFS), Lower Extremity Functional Scale (LEFS), and VAS scale. Satisfaction was assessed on a ten-point scale. The follow-up period was on average 8.61 years (from 7.33 to 10.31). Results: EOTTS had a positive impact on physical activity, and a high rate of patient satisfaction (8.95 ± 1.9) was noted. The treatment led to a reduction in foot pain, as well as an increase in SAAS and LEFS scores (15.6% and 19.3%, respectively, *p* < 0.01). The VAS pain score decreased by 18.6% (*p* < 0.001). SFS and UCLA scores showed a small increase, but it was not statistically significant. A positive correlation was noted between patient satisfaction and time of physical activity per week (R = 0.33, *p* = 0.04), and also between patient satisfaction and SAAS scores (R = 0.43, *p* = 0.005). Pain from other joints (knee, hip) was eliminated or reduced in 40% of patients after surgery. Conclusions: EOTTS with a HyProCure implant is an effective long-term treatment option for partial talotarsal joint dislocation, leading to a reduction in foot pain and increased patient satisfaction, and allowing for a return to physical activity.

## 1. Introduction

The talotarsal joint complex (TTJ) consists of four individual, but kinematically related, joints (posterior, medial and anterior talocalcaneal, and talonavicular). The motion of the talus on the tarsal bones (calcaneus/navicular) is a three-dimensional semi-spiral motion [1,2]. Anatomic functioning of the TTJ depends on the accurate positioning of its articular facets and their co-mobility in all phases of the gait cycle [2,3,4]. Misalignment of any one facet can lead to a change in the distribution of forces in the closed mechanical structure of the TTJ and result in misalignment of the hindfoot. These changes are associated with the occurrence of prolonged pronation (hyperpronation or overpronation) during static and dynamic loading activities, including the stance phase of the gait cycle [3,4]. The etiology of TTJ instability is not fully known. Hypothetical predisposing factors include disorders in the stabilizing ligaments of the talocalcaneal and talonavicular joints, which may be the result of traumatic or non-traumatic causes [5,6]. TTJ instability affects the biomechanical alignment and functioning of the foot and the lower limb, and also the overall gait. It may also affect the proximal musculoskeletal joint alignments (knee, pelvis, and spine) [2,7,8,9,10]. Tarsal pronation is correlated with internal rotation of the tibia and fibula that can cause internal rotational tension in the lower limb and thus lead to increased soft tissue loads and compression forces in other joints of the lower limb, which can result in pain and other associated symptoms. Reports in the literature indicate a relationship between TTJ instability and pain symptoms in the knee [8,9,10,11,12], pelvis [7,8,13], spine [5,9], neck [9], shoulder [9], and even the temporomandibular joint [6,9]. The effects of hyperpronation on foot function have also been noted, including plantar fasciopathy, posterior tibial tendinopathy, hallux valgus, and metatarsal pain, all of which may manifest as foot pain [14,15]. These biomechanical relationships require a consideration of TTJ instability in all patients with secondary symptoms. Therefore, the treatment should be based on stabilization of the TTJ, which will contribute to the overall improvement in the musculoskeletal system’s function, alleviation of pain, and increase in the possibility of practicing sports [16].

Extra-osseous talotarsal stabilization (EOTTS) is a common surgical treatment intended to stabilize and maintain the alignment of the articular facets of the TTJ. A titanium implant, HyProCure^®^ (GraMedica, Macomb, MI, USA) is inserted into the naturally occurring space between the talus and calcaneus, the sinus tarsi, via a minimally invasive procedure (usually a 1–1.5 cm skin incision). For each patient, the size of the implant is selected individually with intraoperative assessment of the degree of correction. The aim of the procedure is to restore the pronation range of the hindfoot, i.e., from three to five degrees [2]. The device prevents the partial dislocation of the talus on the tarsal mechanism while allowing the natural TTJ semi-spiral motion. It is a minimally invasive and conservative alternative to traditional surgical stabilization procedures such as soft tissue augmentation procedures (endoscopic tendon debridement, tenosynovectomy, etc.), medial/lateral column osteotomy, and arthrodesis. EOTTS is associated with a reduced risk of post-operative complications and a shorter recovery time while maintaining the positive effects of treatment [2,10,17,18,19,20]. Therefore, the main working hypothesis in our study was whether physically active patients were able to return to the same level of physical activity after TTJ instability surgeries. The purpose of this prospective study was to monitor activity levels pre- and post-EOTTS in adult patients in the long-term. Most studies only monitor sinus tarsi implant recipients for a short- to mid-term follow-up, and do not report on activity post-surgery. Patient-perceived satisfaction and complications in the long-term are also important. Another aspect of our study was to determine if EOTTS had any effect in reducing pain in other joints. The correlation between hyperpronation of the foot and secondary proximal tissue damage is well established, but there has not been a long-term evaluation to determine if EOTTS could have a positive effect.

## 2. Materials and Methods

Patients treated surgically with EOTTS were enrolled into this prospective study. The inclusion criteria for this study were (1) TTJ displacement on orthopedic examination and radiographic evidence showing a talar 2nd metatarsal > 16 on the weightbearing AP view and/or a talar declination >21 on the lateral view, and (2) an age > 18 years old. Patients with additional or other structural pathologies of the foot and ankle such as hallux limitus/rigidus, hallux valgus–metatarsus primus varus/elevatus, metatarsus adductus, and stage IIB posterior tibial tendonopathy were excluded from the study.

**Figure 1:**
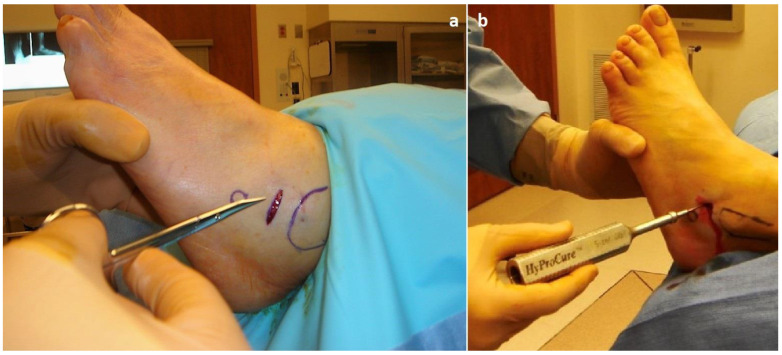
Intraoperative images from the extra-osseous talotarsal stabilization (EOTTS) using HyProCure. (**a**) Minimal-invasive approach with linear skin incision over the sinus tarsi at a distance of 1 cm from the distal aspect of the fibula. (**b**) The trial sizing allows to determine what implant size will provide the most optimal degree of correction.

**Figure 2:**
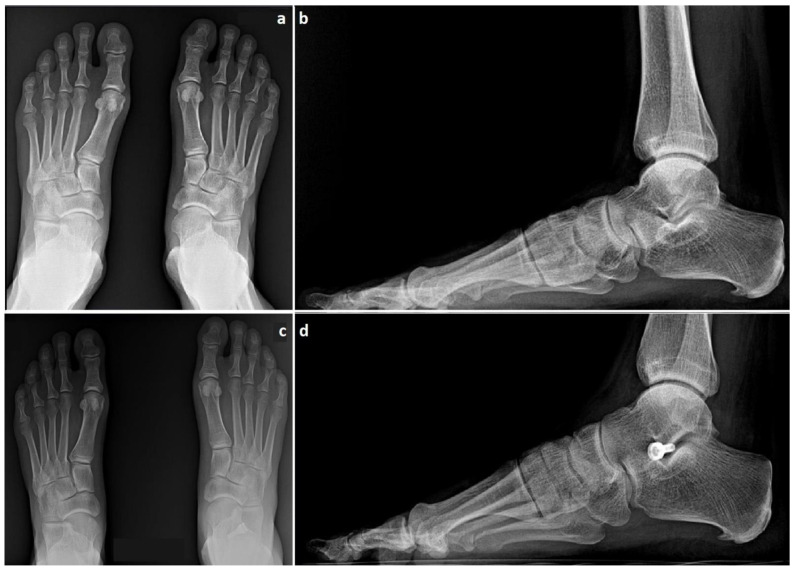
Foot radiographs in a–p (anterior–posterior) and lateral projections before surgery (**a**,**b**) and during the follow-up visit 12 months after surgery (**c**,**d**). All imaging examinations were performed in a standing position.

All patients underwent surgical treatment of TTJ with the HyProCure^®^ EOTTS implant (GraMedica^®^, Macomb, MI, USA) between 2012 and 2015 according to the original surgical technique [2] [Figure 1]. They were followed up with in the outpatient department after 2 and 6 weeks, and then at 6 and 12 months from the initial surgical intervention [Figure 2]. In addition, patients were invited for a final follow-up visit in March 2023. Forty-one adult patients (5 males, 12.2%; 36 females, 87.8%) having a mean age at the time of surgery of 46.4 years (±15.6, median 54) were included. Their average BMI before surgery was 24.2 (±3.2, median 23.95) with an average body weight of 66.6 (±10.5, median 63.5) kilograms. The average final follow-up time was 8.6 years (range: 7.3 to 10.3). All patients agreed to participate through signed written consent in accordance with the World Medical Association’s Declaration of Helsinki. The patients’ physical activity and foot functional scores were assessed in the pre-operative period and during the last follow-up visit using questionnaires—the UCLA Activity Score, Symptom-Related Ankle Activity Scale (SAAS) and Lower Extremity Functional Scale (LEFS) [21]. Furthermore, patients were asked to indicate what sports activities they participate in during the week and how much time they spend on these activities on average during the week. These scores were then matched with the patient’s Sports Frequency Score (SFS). The patients were then asked to rate the level of pain on the Visual Analogue Scale (VAS) that they feel from the affected foot during physical activity. In addition, the pain in other joints reported by patients was recorded. After surgery, during follow-up visits, the complications reported by patients and time to return to physical activity were also recorded. At their last follow-up visit, patients were assessed according to the Maryland Foot Score (MFS). In addition, each patient was asked to determine the degree of satisfaction with the surgical treatment on a ten-point scale (1-very bad, 10-very good) and to determine whether the surgery had a positive impact on the ability to undertake physical activity (Yes/No question).

Statistical analyses were performed using the Statistica 13.0.2 program (StatSoft Polska Sp. z o. o., Kraków, Poland). All comparisons were performed between pre-operative and post-operative results. In accordance with the normality of the distribution determined using the Shapiro–Wilk test, either a student *t*-test or a U Mann–Whitney test were used for dependent samples. Distribution normality was examined using the Shapiro–Wilk test. A correlation matrix was also prepared for the collected data. The significance level was set to a *p* value below 0.05.

## 3. Results

Forty-one patients (62 feet) were qualified for the study. Of this group, 20 patients underwent surgery on one foot, while 21 patients underwent surgery on both feet during two separate operations (the second foot was operated 4 to 6 weeks after the first one). The data distribution on the operated side (left or right) is evenly distributed as 31 vs. 31 feet. The number of patients used for statistical analyses was n = 41. Following EOTTS surgery, patients were able to increase their level of physical activity without pain in their foot or ankle. The SAAS score revealed an average increase of 15.6%. The pre-EOTTS score of 71.7 (±19.99, median 60) jumped to 82.9 (±18.7, median 80) post-EOTTS (*p* = 0.002) [Table 1]. Patients averaged 19.3% higher in their LEFS score after EOTTS: 74.2 (±23.7, median 80.3) was recorded pre-operatively, and 88 post-operatively (±15.3, median 95) (*p* = 0.0006). Along with the improvement in foot and ankle function, patients also reported a decrease in pain on the VAS by an average of 18.6% after surgery: 4.6 (±3.3, median 5) pre-operatively, compared to 0.9 (±1.5, median 0) post-operatively (*p* < 0.0001) [Table 2]. Most of the patients (n = 29, 70.7%) were absolutely pain-free (VAS = 0), ten patients (24.4%) had mild pain (VAS = 1–3), and one patient (2.4%) had moderate pain (VAS = 4) at the last follow-up. One patient (2.4%) experienced severe pain (VAS = 6) after the surgery. The mean MFS score at the last follow-up was 98 (±2.9, median 100). The distribution of the average results of individual fields in the MFS is as follows: pain—44.2 (±1.9, range: 40 to 45); distance walked—9.9 (±0.4, range: 8 to 10); stability—3.9 (±0.3, range: 3 to 4); support—4 (±0); limp—3.95 (±0.2, range: 3 to 4); shoes—9.6 (±1.1, range: 7 to 10); stairs—3.9 (±0.3, range: 3 to 4); terrain—3.7 (±0.7, range: 2 to 4); cosmesis—9.95 (±0.3, range: 8 to 10); motion—5 (±0, range: 5). Patients returned to physical activity, on average, 3.4 (±1.80, median 3) months after surgery.

**Figure 3:**
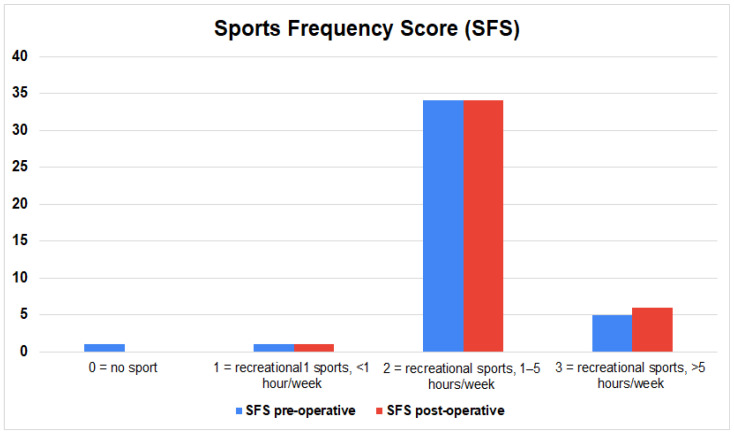
Result from the Sports Frequency Score (SFS) before and after surgery. Recreational sport was defined as sport for fitness without intention to win.

**Figure 4:**
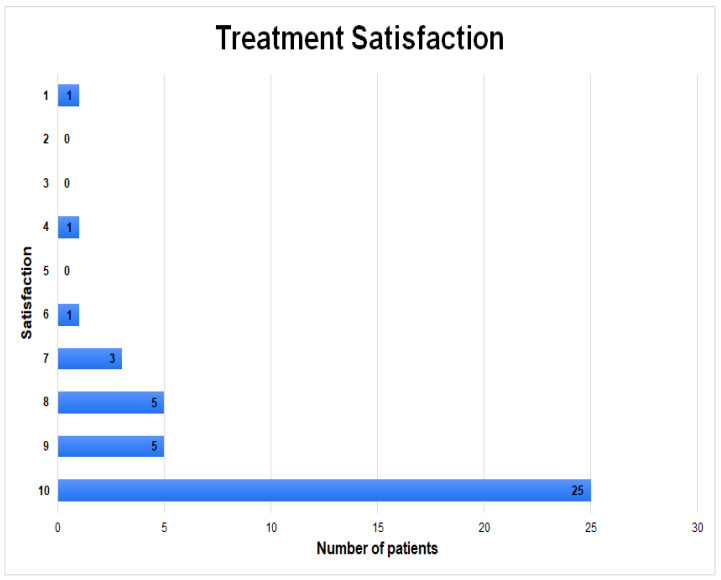
Distribution of results on a ten-point scale of patients’ satisfaction with surgical treatment (1-very dissatisfied, 10-very satisfied).

The most common activities before and after surgery were walking (n = 19, 46.3%), cycling (n = 17, 41.4%), swimming (n = 7, 17.1%), and running (n = 6, 14.6%). All patients who qualified for the study remained active and performed at least one physical activity a week after surgery. In total, before surgery, patients spent time on 79 different forms of physical activity, while after surgery this result decreased to 73 (*p* = 0.3723) [Table 3]. Patients before surgery had about 1.95 (±1.05, median 2) activities per week, while after surgery this result decreased by 6.6%, and was 1.8 (±1, median 2) (*p* = 0.372). However, the amount of time spent in physical activity increased by an average of 11.1% from 153.7 (±126.3, median 120) minutes per week pre-operatively to 170 (±102, median 130) minutes after surgery (*p* = 0.145). The Sports Frequency Score (SFS) showed a trend indicating that patients maintained the same level of physical activity after surgery—the average score before surgery was 2.1 (±0.5, median 2), and after surgery 2.1 (±0.4, median 2) (*p* = 0.1088) [Figure 3]. There was also a non-significant but slight increase in the UCLA score from 6 (±2.7, median 6) pre-operatively to 6.1 (±2.5, median 6) post-operatively (*p* = 0.787). According to the subjective feelings of patients, 30 (73.2%) of them declared that the surgical treatment had a positive impact on the possibility of undertaking physical activity. A high rate of satisfaction with the surgical treatment was also noted, which averaged 8.95 (±1.9) on a ten-point scale [Figure 4].

Minor complications were noted in two cases (4.9%). One patient had a wound healing issue (n = 1, 2.4%) that self-resolved without any negative impact on the outcome. Another patient experienced persistent pain after six months and that continued through to their last follow-up (8 years from surgery) and also limited their activities (n = 1, 2.4%). There were no implant-related removal or revision surgeries. Before surgery, 25 patients (61%) reported pain in other joints—knee, hip, and the lumbar spine. After the surgical treatment, pain in these other areas was eliminated (n = 4, 10%) or reduced (n = 6, 15%) in some patients. In the rest of the patients from this group, it was not observed that surgical treatment reduced pain in other joints. 

The correlation matrix showed a positive correlation between the total Maryland Foot Score and post-operative SAAS scores (R = 0.3241, *p* = 0.039). In addition, the total score of the MFS scale shows a negative correlation in the intensity of pain in patients after surgery, expressed in VAS (R = −0.4427, *p* = 0.004). The results obtained in the ten-point scale of satisfaction with surgical treatment correlated positively with the following items of the Maryland Foot Score: distance walked (R = 0.4231, *p* = 0.006), limp (R = 0.4231, *p* = 0.006), and stairs (R = 0.4996, *p* = 0.001) [Table 4]. Next, a correlation matrix was made between the degree of patient satisfaction and the level of physical activity. A positive correlation was found between the level of satisfaction and time of physical activity per week (R = 0.3261, *p* = 0.040) and SAAS scores (R = 0.4317, *p* = 0.005). The VAS and UCLA scales did not correlate statistically significantly with the level of patient satisfaction (*p* > 0.1).

## 4. Discussion

The goal of our study was to document a return to physical activity after surgical treatment of TTJ instability with an EOTTS implant in adults. We found that patients were able to perform moderate to strenuous activities with no foot symptoms. This is confirmed by the results obtained in the Symptom-Related Ankle Activity Scale (SAAS), where an average improvement of 15.6% was noted. There have been multiple studies showing triplane radiographic normalization of pre-EOTTS pathologic angles, and positive functional outcome scores, but there was a lack of research on returning to physical activity after EOTTS with the use of a HyProCure implant [19,20,22]. For example, Martinelli et al. showed that 91.8% of pediatric patients (mean age of the patients at the time of surgery—10.7 years) were able to play sports after subtalar arthroeresis [23]. In another pediatric study, Herdea et. al. also studied the ability of pediatric patients to sprint 100 m. They found that patients treated with a sinus tarsi implant were able to run longer distances than the control group of untreated pediatric patients [24]. Our study had a minimum of 7 years of follow-up in adult patients, 100% of whom were able to participate in the sports activity of their choice. Another comparison, in the cases of realignment surgery due to varus and valgus ankle osteoarthritis, patients were able to perform at a higher activity level without symptoms after surgical realignment. Pagenstert et al. in their study also observed a significant decrease in the number of patients who were unable to perform daily activities without pain before surgery from 60% to 8.6% after surgery (*p* = 0.0001). The number of patients who were able to perform moderate and strenuous work without pain also increased from 0% to 48.6% post-operatively [21]. In our study, patients were treated before degenerative changes developed due to TTJ instability, and they did not report pain that prevented them from performing daily activities. We have observed an increase in the number of patients performing moderate work (recreational jogging, skiing, cycling) without pain from 26.8% to 34.1% post-operatively, and in the group performing strenuous work without pain, we observed an increase from 22% to 44% after surgery. The reduction in the pain level was also noted in the VAS, where patients reported pain, on average, at the level of 4.6 points before surgery, and 0.9 points after surgery. Similar results were reported by Pin Feng et al. among patients treated with EOTTS, where the VAS score decreased from 4.2 to 1.4 [25].

After a mean follow-up period of 8.6 years, all patients showed very good functional results presented in both the MFS (mean score = 98) and LEFS (mean score = 88.6) scales. In comparison, Graham et al. in their study, in a group of 78 adult patients treated with the HyProCure^®^ implant, recorded an average MFS score of 88 [2]. In another study conducted by Chen et al., 69 pediatric flexible flatfoot patients (107 feet) treated with the HyProCure^®^ implant reported a mean MFS score of 90.4 after surgery [26]. As noted by Chen et al., improper trial sizing could lead to a larger size selection, thus preventing the ideal depth of implant placement, which is a risk factor for unsatisfactory post-operative efficacy. The vast majority of our patients after surgery were able to perform their daily activities without any major difficulties. As noted by Kheyrandish et al., flexible flat foot as an abnormality can lead to poor performance in functional tests and sport activities [27]. It can therefore be concluded that surgical treatment aimed at normalizing hindfoot pronation leads to an increase in efficiency and functional stability, and consequently results in an increase in the level of physical activity. We also noted a positive correlation between the MFS and post-operative SAAS scale and a negative correlation between the MFS and post-operative VAS. Thus, it can be assumed that, along with the reduction in pain, there is an increase in physical activity, which leads to an increase in the functional results of the foot. Shannon L Merkle et al. showed the connection between regular physical activity, improved mobility, and reduced pain levels [28]. We indicated a positive correlation between patients’ satisfaction with surgical treatment and both SAAS scores and the time spent on a physical activity. In addition, the results from the MFS scale in the disciplines: distance walked, limp, and stairs, showed a positive correlation with the level of patient satisfaction. Patients’ satisfaction with surgical treatment increases also with the reduction in pain during activities and with the increase in functional results and physical activity, which was observed by other authors [29]. This is also confirmed by the patients in our study, where 73.2% declared that the surgical treatment had a positive impact on the possibility of undertaking physical activity, with an average rating of the surgery at 8.95. Similar results were reported by Ciechanowicz et al. in a study about the return to physical activity after a scarf osteotomy for hallux valgus, where 77% of patients declared that they were satisfied with the outcome of the surgery and physical activity was less difficult for them after the surgery, with an average rating of 8.2 [30]. In our study, most of the patients were able to maintain and often increase their level of physical activity after surgery (100% of patients were able to perform recreational sport). The amount of time spent on physical activity increased by an average of 11.1%. On the other hand, the results from the Sports Frequency Score confirm that patients maintained the same level of activity before surgery with a median score 2, both pre- and post-operatively. In addition, we observed a similar level of physical activity, measured with the UCLA activity score—6.02 pre-operatively and 6.09 post-operatively. For comparison, Martinelli et al. showed an increase in the UCLA score from 2.6 to 6.1 after a medializing calcaneal osteotomy and navicular–cuneiform arthrodesis for relieving the symptomatic effects of flat feet. They also showed that 36.6% of patients after surgery managed to return to low sports activities, 40% to moderate, and 23.3% to intensive [31]. On another hand, after surgical treatment of the hallux valgus, an increase in the average UCLA score from 6.24 to 6.53 was observed. In addition, the same study observed an increase in the amount of time spent on physical activity by about 19% after surgery [30]. Fuller et al. showed in their study that 2.4% of patients were sedentary with no physical activity, 31.7% were moderately active, 29.3% were highly active, and 36.6% were extremely active after surgical treatment of flatfoot [32]. Additionally, in our study, we observed that after surgery most patients were able to practice recreational sports at the level of 1–5 h/week (83%) and more than 5 h/week (15%). Fuller et al. reported that the most frequently chosen activities among patients were walking, biking, and swimming and none of the activities were interrupted after the surgery [32]. Similar results were noted in our study and the main activities taken by patients were walking, cycling, swimming, and running. 

Moreover, deformities in the foot lead to various musculoskeletal symptoms in the lower limb, such as knee or hip pain. Kolodziej et al. showed that EOTTS can reduce in vivo forces in the medial knee compartment. This helps illustrate the importance of rearfoot alignment and stabilization in preventing and treating chronic knee pain [10]. Rao et al. indicated that an aberrant foot structure has been linked to foot osteoarthritis, as well as osteoarthritis and pain at the knee and hip [33]. As shown by Gross et al. in their study, abnormal forefoot alignment may be associated with ipsilateral hip pain or tenderness and the need for total hip arthroplasty in future [34]. Additionally, in our study, we observed the coexistence of problems with other joints. About 60% of patients with TTJ instability reported pain in other joints before surgery—most often in the knee, and hip, but also in the temporomandibular joint and in the lumbar spine. After surgery, pain in other joints subsided or decreased in 40% of patients that had pain in these joints prior to surgery (10/25). These results indicate that previously implemented treatment of foot instability may contribute to the improvement in the function of other joints and reduce the pain reported by patients. In our study, during the follow-up, persistent pain was present only in one case (2.4%). The pain persisted for eight years after the surgery and limited the patient’s daily functioning. The patient required occasional pain medication, but the implant was not removed because the patient did not consent to re-operation. The literature indicates a complication rate and the need to remove the implant (HyProCure) of 0–20.6%, most often due to sinus tarsi pain [2]. As Ålund points out in his study, the size of the implant is most responsible for chronic pain after surgery [35]. An implant size that is too small can lead to under-correction and allow continued subtalar instability, while too large of a size may lead to “over-stuffing” and prevent a central placement of the implant. In another study, Bresnahan et al. reported no clinically significant post-operative complications for 46 feet in 35 patients. Only two patients (two feet, 4.35%) had their implants removed due to lack of symptom relief after HyProCure implementation (one patient) and discomfort during walking and physical activity (one patient) [36]. In our study, reoperation and removal of the implant were not performed. These results indicate that EOTTS treatment has a relatively low rate of complications; the most common complication is pain, which may be caused by the wrong size of the implant.

One of the limitations of our study is the lack of a control group. However, the aim of our study was to determine the possibility of returning to physical activity in patients practicing recreational sports. In addition, the deformity of the other foot may also have had a negative impact on the level of physical activity. However, over half of the patients had surgery on both feet, which reduces this limitation of our study. Other studies have already compared patients with EOTTS surgery to untreated patients, or to other types of surgical correction; we felt it was unnecessary to once again show that patients with EOTTS are able to be more active than untreated patients. Likewise, we did not obtain pre- and post-EOTTS radiographic measurements as many other papers have already established the fact that EOTTS can normalize pre-EOTTS pathologic radiographic angles. The diversity of our study group could reduce reliability of our results, as the patients represented various sports and training levels. Nevertheless, we found that regardless of the type and level of training after EOTTS, a return to physical activity was possible in the vast majority of patients. In our study we observed a trend indicating an increase in the ability of patients to undertake physical activity with a simultaneous decrease in pain compared to their pre-operative status.

## 5. Conclusions

The surgical treatment of talotarsal joint instability with the use of an EOTTS implant can help provide the patient with a more efficient and functioning foot. It has been shown to reduce foot pain and allow patients to return to physical activity. In addition, EOTTS recipients relayed reduced pain in other proximal joints. This study further establishes the safety, efficacy, and increased quality of life and functionality following intervention with EOTTS in adult patients. The overall benefits of EOTTS outweigh any potential complications.

## Figures and Tables

**Table 1:** Results from the Symptom-Related Ankle Activity Scale (SAAS) before and after surgery. ADL-activity of daily living.

Symptom-Related Ankle Activity Scale (SAAS)	Pre-Operative (n, %)	Post-Operative (n, %)
0 = no ADL, disabled by ankle	0 (0%)	0 (0%)
20 = symptoms with ADL, not disabled; sedentary work and walking on even ground possible	1 (2.4%)	0 (0%)
40 = no symptoms with ADL; light work (e.g., nursing), swimming, walking on uneven ground possible	3 (7.3%)	3 (7.3%)
60 = no symptoms with light work; moderate work (e.g., truck driving, heavy domestic work), recreational jogging, skiing, cycling possible	17 (41.5%)	6 (14.6%)
80 = no symptoms with moderate work; strenuous work (e.g., building, forestry), jogging on uneven ground, competitive cycling, skiing possible	11 (26.8%)	14 (34.1%)
100 = no symptoms with strenuous work, all recreational and competitive sports possible (e.g., soccer)	9 (22.0%)	18 (44.0%)

**Table 2:** Comparison of the level of physical activity and the level of pain of patients in the period before and after surgery. SD-standard deviation; UCLA-University of California at Los Angeles; SAAS-Symptom-Related Ankle Activity Scale; LEFS-Lower Extremity Functional Scale; VAS-Visual Analogue Scale.

	Pre-Operative	Post-Operative	*p* Value
Number of physical activities [x/week], mean (SD)	1.95 (±1.1)	1.83 (±1.0)	0.372
Time of physical activity [min/week], mean (SD)	153.7 (±126.3)	170 (±102)	0.145
UCLA activity score, mean (SD)	6.02 (±2.7)	6.09 (±2.5)	0.787
SAAS, mean (SD)	71.7 (±20.0)	82.9 (±18.7)	0.002
LEFS, mean (SD)	74.2 (±23.7)	88.6 (±15.3)	0.0006
VAS, mean (SD)	4.6 (±3.3)	0.9 (±1.5)	<0.0001

**Table 3:** Reported sports activities before and after surgery. In the right column, the distribution of disciplines in terms of the degree of load on the joints of the lower limb are noted.

Physical Activity	Pre-Operative	Post-Operative	Change	Joint-Stress Activities
Walking	19	19	0	Low
Cycling	17	17	0	Low
Swimming	7	7	0	Low
Running	6	6	0	High
Weightlifting	6	7	+1	High
Fitness	6	4	−2	High
Nordic Walking	4	4	0	High
Skiing	4	3	−1	High
Dance	2	2	0	High
Volleyball	2	1	−1	High
Mountain trekking	1	0	−1	High
Go-karts	1	0	−1	Low
Canoeing	1	0	−1	Low
Climbing	1	1	0	High
Martial arts	1	1	0	High
Squash	1	1	0	High

**Table 4:** The table presents the correlation matrix between the Maryland Foot Scale (MFS) and post-operative results from the physical activity and functional questionnaires.

	Time of Physical Activity [min/week],	Number of Physical Activities [x/week]	UCLA	SAAS	VAS	Satisfaction	LEFS
MFS score	0.2203	0.0981	0.0862	0.3241	−0.4427	0.2552	−0.1240
	*p* = 0.166	*p* = 0.542	*p* = 0.592	*p* = 0.039	*p* = 0.004	*p* = 0.107	*p* = 0.440
Pain	0.2573	0.0287	−0.1125	0.1418	−0.5228	0.1284	−0.1203
	*p* = 0.104	*p* = 0.859	*p* = 0.484	*p* = 0.377	*p* = 0.000	*p* = 0.424	*p* = 0.454
Distance walked	0.2248	0.1687	0.0089	0.1581	−0.0982	0.4231	0.0603
	*p* = 0.158	*p* = 0.292	*p* = 0.956	*p* = 0.323	*p* = 0.541	*p* = 0.006	*p* = 0.708
Stability	0.0245	0.0096	0.1120	0.0520	0.0780	−0.0532	0.1346
	*p* = 0.879	*p* = 0.953	*p* = 0.486	*p* = 0.747	*p* = 0.628	*p* = 0.741	*p* = 0.402
Support	--	--	--	--	--	--	--
	*p* = ---	*p* = ---	*p* = ---	*p* = ---	*p* = ---	*p* = ---	*p* = ---
Limp	0.2248	0.1687	0.0089	0.1581	−0.0982	0.4231	0.0603
	*p* = 0.158	*p* = 0.292	*p* = 0.956	*p* = 0.323	*p* = 0.541	*p* = 0.006	*p* = 0.708
Shoes	0.0137	−0.0209	0.0995	0.3637	−0.1795	0.1759	−0.1324
	*p* = 0.932	*p* = 0.897	*p* = 0.536	*p* = 0.019	*p* = 0.261	*p* = 0.271	*p* = 0.409
Stairs	0.1487	0.0305	0.3123	0.4492	−0.0276	0.4996	−0.0439
	*p* = 0.353	*p* = 0.850	*p* = 0.047	*p* = 0.003	*p* = 0.864	*p* = 0.001	*p* = 0.785
Terrain	−0.0404	0.1270	0.2367	0.1117	−0.0921	−0.1227	−0.0233
	*p* = 0.802	*p* = 0.429	*p* = 0.136	*p* = 0.487	*p* = 0.567	*p* = 0.445	*p* = 0.885
Cosmesis	−0.1099	0.1178	0.1969	−0.1458	0.0905	−0.0042	−0.1195
	*p* = 0.494	*p* = 0.463	*p* = 0.217	*p* = 0.363	*p* = 0.573	*p* = 0.979	*p* = 0.457
Motion	--	--	--	--	--	--	--
	*p* = ---	*p* = ---	*p* = ---	*p* = ---	*p* = ---	*p* = ---	*p* = ---

UCLA-University of California at Los Angeles; SAAS-Symptom-Related Ankle Activity Scale; LEFS-Lower Extremity Functional Scale; VAS-Visual Analogue Scale.

## Data Availability

Not applicable.
